# Supplementary material for: A Likelihood Approach for Real-Time Calibration of Stochastic Compartmental Epidemic Models
Source: PLoS Comput Biol. 2017 Jan 17;13(1):e1005257. doi: 10.1371/journal.pcbi.1005257 (PMC5240920; doi:10.1371/journal.pcbi.1005257)
Supplement: S1 File — (TAR.GZ) [file pcbi.1005257.s014.tar.gz › HSPH_Online-SI-Revision/MSS10/n10-severe/n10-severe_NEW_table-4.pdf]

1	R0	{1.68952, 11.8402, 1.}	{1.60394, 9.21197, 1.42}	{{1.24751, 3.07157, 0.}, {1.7434, 11.5667, 2.}}	{9.59389, 22.1975, 100.}	inf	719.	576.861	{172.347, 759.925}	24.0053	0.183766	newly	473.	469.58	{387., 520.}	9.4926	0.0457895	Reff	1.34131	1.29084	{1.06013, 1.38786}	8.04306	ar	6872.	6100.05	{3582., 7224.}	15.5755
2	R0	{1.71739, 9.62661, 0.1}	{1.48878, 7.737, 1.35}	{{1.24751, 3.07157, 0.}, {2.12588, 19.2116, 4.}}	{21.5263, 62.6129, 1350.}	inf	504.	363.551	{121.361, 948.997}	59.3557	0.369314	newly	426.	372.7	{289., 455.}	17.1925	0.0911538	Reff	1.47352	1.28955	{1.11712, 1.71903}	18.5117	ar	6874.	5184.72	{3505., 8132.}	32.492
3	R0	{1.48936, 6.23975, 0.1}	{1.29631, 3.97152, 0.12}	{{1.22544, 3.07157, 0.}, {1.44485, 6.00596, 0.}}	{13.9547, 39.3647, 204.}	inf	371.	230.094	{138.795, 336.35}	41.5834	0.25203	newly	491.	443.93	{323., 578.}	18.4745	0.0924152	Reff	1.29008	1.14009	{1.0872, 1.24784}	12.4129	ar	5666.	4133.54	{3381., 5239.}	28.0049
4	R0	{1.62394, 7.51135, 1.}	{1.24663, 3.18339, 0.01}	{{1.22544, 2.5364, 0.}, {1.25474, 3.26445, 0.}}	{23.234, 57.6189, 99.}	inf	401.	170.033	{100.132, 232.515}	57.5977	0.400766	newly	435.	418.78	{261., 580.}	25.0207	0.120757	Reff	1.42192	1.12034	{1.09782, 1.13623}	21.2098	ar	6417.	3686.15	{3221., 4115.}	42.5565
5	R0	{1.53632, 8.57652, 1.}	{1.26124, 3.20708, 0.08}	{{1.22544, 2.5364, 0.}, {1.28596, 3.98513, 0.}}	{17.9051, 62.6063, 96.}	inf	485.	198.183	{101.597, 277.988}	59.1375	0.416402	newly	460.	478.4	{310., 664.}	21.8783	0.0936348	Reff	1.29543	1.09948	{1.06938, 1.12108}	15.1259	ar	6260.	3827.49	{3228., 4430.}	38.858
6	R0	{1.47166, 5.7434, 0.1}	{1.23812, 3.00772, 0.}	{{1.22544, 2.5364, 0.}, {1.25474, 3.26445, 0.}}	{16.713, 51.8044, 100.}	inf	232.	119.929	{36.1811, 193.25}	54.2618	0.405729	newly	328.	321.36	{101., 524.}	39.2073	0.213971	Reff	1.32906	1.13155	{1.11099, 1.1529}	15.5426	ar	5650.	3431.63	{2518., 3976.}	40.5173
7	R0	{1.48179, 8.87369, 0.1}	{1.25673, 3.51254, 0.11}	{{1.22544, 3.07157, 0.}, {1.28596, 3.98513, 0.}}	{15.1881, 60.4162, 194.}	inf	510.	193.973	{115.196, 265.76}	61.9662	0.445891	newly	397.	381.28	{249., 529.}	23.5466	0.11435	Reff	1.14379	1.00972	{0.980947, 1.03339}	11.7215	ar	5233.	3615.2	{2889., 4170.}	30.9153
8	R0	{1.53669, 6.47807, 0.1}	{1.82239, 10.5214, 1.67}	{{1.47651, 6.00596, 1.}, {1.94282, 12.1446, 2.}}	{20.3553, 66.387, 1584.}	inf	392.	639.891	{401.225, 767.739}	67.423	0.220471	newly	477.	500.03	{440., 552.}	8.61845	0.0360504	Reff	1.32417	1.52242	{1.27535, 1.61706}	16.5828	ar	5934.	7246.68	{5756., 7881.}	24.7516
9	R0	{1.57227, 6.20989, 0.1}	{1.70047, 8.08007, 1.26}	{{1.24751, 2.5364, 0.}, {2.46976, 17.5057, 3.}}	{31.5043, 95.6306, 1274.}	inf	215.	179.335	{20.0772, 374.289}	63.4114	0.434989	newly	311.	211.86	{91., 298.}	39.9421	0.262327	Reff	1.49381	1.614	{1.19255, 2.3131}	29.955	ar	6136.	5623.41	{3126., 8915.}	41.4496
10	R0	{1.54936, 6.50938, 0.1}	{1.84086, 7.20499, 0.66}	{{1.24751, 2.5364, 0.}, {2.86118, 15.5722, 2.}}	{32.2598, 72.3003, 694.}	inf	423.	541.489	{135.715, 1209.88}	82.5294	0.316118	newly	555.	646.95	{442., 820.}	27.836	0.114641	Reff	1.36483	1.57276	{1.12568, 2.28662}	26.4134	ar	6275.	6350.7	{3806., 9292.}	29.6328
11	R0	{1.42179, 3.18601, 0.1}	{1.29027, 2.36565, 0.02}	{{1.24751, 1.89883, 0.}, {1.39162, 2.5364, 0.}}	{9.30086, 25.7488, 116.}	inf	124.	109.107	{26.7236, 182.859}	40.0321	0.259994	newly	375.	456.46	{111., 783.}	59.184	0.277282	Reff	1.33549	1.21383	{1.16628, 1.31182}	9.14074	ar	5322.	3873.85	{3076., 5062.}	27.2937
12	R0	{1.48035, 6.46183, 0.1}	{1.65928, 7.75999, 1.16}	{{1.24751, 2.5364, 0.}, {2.22867, 15.6411, 3.}}	{25.7876, 83.945, 1182.}	inf	191.	239.965	{68.1582, 474.747}	81.8713	0.326063	newly	266.	287.63	{197., 403.}	22.5226	0.0938688	Reff	1.39049	1.54263	{1.18178, 2.037}	23.7292	ar	5793.	5883.25	{3479., 8550.}	33.598
13	R0	{1.75755, 8.53854, 1.}	{1.489, 4.61969, 0.}	{{1.24751, 3.07157, 0.}, {1.47608, 4.06661, 0.}}	{22.0064, 59.2979, 100.}	inf	456.	244.219	{130.139, 298.195}	57.9272	0.364956	newly	444.	487.4	{344., 634.}	24.4099	0.101805	Reff	1.57195	1.35752	{1.15053, 1.36023}	19.3954	ar	6832.	5348.4	{3788., 5905.}	25.445
14	R0	{1.47355, 4.89451, 0.1}	{1.75161, 6.7386, 0.27}	{{1.44762, 3.83841, 0.}, {2.60786, 15.5255, 1.}}	{19.7647, 65.2883, 322.}	inf	339.	475.545	{227.881, 1057.48}	61.6468	0.183112	newly	540.	659.39	{500., 832.}	26.5389	0.100623	Reff	1.31367	1.52243	{1.29263, 2.13875}	16.7157	ar	5710.	6488.2	{5379., 9059.}	17.2434
15	R0	{1.62071, 5.98665, 0.1}	{1.24164, 2.84916, 0.}	{{1.22544, 2.5364, 0.}, {1.25474, 3.26445, 0.}}	{23.389, 52.4081, 100.}	inf	269.	96.0704	{26.507, 163.001}	64.2861	0.55175	newly	451.	291.65	{76., 515.}	41.7894	0.321896	Reff	1.51245	1.18015	{1.16027, 1.19716}	21.9709	ar	6635.	3494.2	{2706., 4074.}	47.3369
16	R0	{1.67268, 7.7256, 1.}	{1.92471, 9.897, 1.63}	{{1.24751, 2.5364, 0.}, {2.57305, 16.884, 3.}}	{38.4436, 89.7134, 155.}	inf	657.	893.102	{137.202, 1542.85}	98.4019	0.434712	newly	678.	680.16	{425., 797.}	16.9145	0.0857657	Reff	1.34935	1.46739	{1.05465, 1.85607}	28.329	ar	6947.	6491.29	{3351., 9028.}	37.3129
17	R0	{1.67805, 11.1417, 1.}	{1.29138, 4.00819, 0.12}	{{1.22544, 3.07157, 0.}, {1.40191, 5.7404, 1.}}	{23.0426, 64.0253, 90.}	inf	455.	169.76	{89.9139, 250.439}	62.6901	0.456473	newly	326.	334.18	{201., 457.}	24.2822	0.109093	Reff	1.48021	1.17572	{1.12016, 1.26757}	20.5703	ar	6625.	4077.99	{3286., 5101.}	38.4454
18	R0	{1.66743, 9.99046, 0.1}	{1.26185, 3.51743, 0.07}	{{1.22544, 3.07157, 0.}, {1.28596, 3.98513, 0.}}	{24.3238, 64.7921, 156.}	inf	487.	161.841	{90.3151, 227.623}	66.7677	0.509394	newly	415.	358.9	{209., 467.}	20.3422	0.110238	Reff	1.42932	1.12259	{1.08995, 1.14515}	21.46	ar	6899.	3760.22	{3130., 4376.}	45.4962
19	R0	{1.70787, 9.85478, 0.1}	{1.2884, 3.99911, 0.14}	{{1.28596, 3.98513, 0.}, {1.28596, 3.98513, 0.}}	{24.5613, 59.4196, 224.}	inf	650.	269.429	{202.049, 328.969}	58.5493	0.389129	newly	499.	500.06	{394., 588.}	12.2084	0.054449	Reff	1.37449	1.0859	{1.07622, 1.09366}	20.9962	ar	6867.	4197.98	{3819., 4530.}	38.8673
20	R0	{1.53809, 10.5848, 1.}	{1.23423, 3.18841, 0.05}	{{1.22544, 2.5364, 0.}, {1.25474, 3.26445, 0.}}	{19.7557, 69.8774, 99.}	inf	420.	123.824	{55.3842, 195.92}	70.518	0.576362	newly	300.	303.11	{150., 457.}	32.7033	0.155553	Reff	1.34152	1.11294	{1.09857, 1.14321}	17.0387	ar	6108.	3363.3	{2549., 4017.}	44.9361
21	R0	{1.59925, 6.74479, 0.1}	{1.50008, 4.57181, 0.27}	{{1.24751, 2.5364, 0.}, {1.53501, 4.06661, 0.}}	{16.8263, 53.9602, 352.}	inf	387.	271.934	{119.97, 329.143}	54.1249	0.30935	newly	488.	522.97	{319., 713.}	24.998	0.111819	Reff	1.42989	1.35154	{1.14098, 1.38801}	14.4248	ar	6411.	5307.93	{3635., 6008.}	24.4413
22	R0	{1.38779, 4.40977, 0.1}	{1.48556, 7.56477, 2.58}	{{1.06836, 1.81005, 0.}, {1.92096, 15.109, 5.}}	{24.4967, 127.407, 2546.}	inf	183.	296.447	{40.8033, 591.609}	116.091	0.413875	newly	337.	338.77	{158., 500.}	34.6202	0.161373	Reff	1.26761	1.33144	{0.985093, 1.68419}	21.7204	ar	5154.	4845.94	{1360., 7838.}	47.3112
23	R0	{1.53657, 7.50352, 1.}	{1.26259, 3.45198, 0.06}	{{1.22544, 3.07157, 0.}, {1.28596, 3.98513, 0.}}	{17.8304, 53.9952, 98.}	inf	441.	172.756	{90.5483, 252.668}	60.8264	0.441872	newly	429.	382.87	{202., 542.}	26.1841	0.135959	Reff	1.31868	1.11731	{1.08713, 1.13812}	15.2711	ar	5928.	3759.68	{3096., 4357.}	36.5776
24	R0	{1.71131, 6.24526, 0.1}	{1.36775, 3.35395, 0.}	{{1.24751, 2.5364, 0.}, {1.47488, 4.06661, 0.}}	{20.1102, 46.296, 100.}	inf	217.	122.039	{34.3475, 199.131}	44.6771	0.326343	newly	358.	339.97	{122., 506.}	34.9022	0.190597	Reff	1.60743	1.29744	{1.18029, 1.40662}	19.321	ar	6924.	4667.8	{3372., 5830.}	32.5916
25	R0	{1.4412, 3.07729, 0.1}	{1.34665, 2.86515, 0.01}	{{1.24751, 2.19254, 0.}, {1.53501, 4.02511, 0.}}	{11.3283, 20.4533, 108.}	inf	56.	43.0789	{14.6238, 72.5513}	39.7008	0.263341	newly	207.	160.64	{54., 280.}	46.8019	0.302758	Reff	1.41454	1.32332	{1.22334, 1.51384}	11.2077	ar	5810.	4369.11	{3212., 6290.}	29.0015
26	R0	{1.4159, 4.93858, 0.1}	{2.15419, 10.2604, 1.33}	{{1.24751, 2.5364, 0.}, {2.688, 14.8279, 2.}}	{55.959, 130.635, 1304.}	inf	225.	603.313	{132.923, 900.646}	185.583	0.420355	newly	397.	532.46	{348., 639.}	39.1537	0.142456	Reff	1.30079	1.87787	{1.15743, 2.30318}	47.9654	ar	5255.	7489.35	{3838., 9178.}	51.3505
27	R0	{1.57465, 7.05414, 0.1}	{1.45899, 2.96295, 0.24}	{{1.24751, 1.89883, 0.}, {1.39162, 2.5364, 0.}}	{16.1044, 75.3456, 320.}	inf	216.	119.621	{25.5961, 224.923}	59.7236	0.454375	newly	292.	570.03	{191., 1087.}	113.332	0.303163	Reff	1.4934	1.39518	{1.20377, 1.34918}	14.8291	ar	6359.	5164.72	{3800., 5466.}	24.6102
28	R0	{1.41422, 4.62402, 0.1}	{2.51896, 12.6816, 0.17}	{{2.22671, 10.7276, 0.}, {2.96947, 15.963, 1.}}	{78.1166, 174.254, 236.}	inf	138.	429.986	{350.067, 505.82}	211.584	0.488792	newly	285.	343.88	{287., 396.}	21.0456	0.0803474	Reff	1.35298	2.3353	{2.08043, 2.72488}	72.6038	ar	5485.	8909.5	{8530., 9362.}	62.4339
29	R0	{1.41901, 3.63017, 0.1}	{1.63828, 4.71164, 0.08}	{{1.24751, 2.5364, 0.}, {2.59708, 9.75224, 0.}}	{22.6934, 47.4679, 166.}	inf	127.	177.579	{52.6979, 422.266}	70.4232	0.235834	newly	382.	374.97	{153., 550.}	32.0026	0.159344	Reff	1.3631	1.55963	{1.19819, 2.42265}	21.5111	ar	5979.	5914.14	{3770., 9034.}	23.2624
30	R0	{1.60719, 4.72984, 0.1}	{2.62453, 10.2498, 0.36}	{{2.26582, 8.12562, 0.}, {2.91001, 12.0293, 1.}}	{63.464, 116.985, 392.}	inf	120.	249.368	{186.074, 310.067}	109.235	0.311958	newly	274.	293.61	{208., 370.}	18.2591	0.0760507	Reff	1.56171	2.51511	{2.17891, 2.76685}	61.2023	ar	6479.	8988.65	{8481., 9338.}	38.9213
31	R0	{1.38175, 6.26468, 0.1}	{1.44348, 5.26281, 0.95}	{{1.24751, 2.5364, 0.}, {2.43839, 19.0531, 5.}}	{21.4557, 83.7133, 1008.}	inf	357.	326.556	{78.3417, 1189.62}	88.0162	0.443774	newly	432.	479.84	{235., 661.}	32.8981	0.151383	Reff	1.2035	1.24408	{1.10089, 1.91511}	16.809	ar	5397.	4423.59	{2869., 8845.}	39.8501
32	R0	{1.4947, 8.8847, 1.}	{1.25035, 3.42428, 0.11}	{{1.22544, 3.07157, 0.}, {1.28596, 3.98513, 0.}}	{16.3478, 61.4586, 93.}	inf	462.	188.463	{125.017, 243.799}	59.2072	0.409316	newly	361.	384.94	{273., 490.}	20.8809	0.0940812	Reff	1.18634	1.02658	{0.999656, 1.05604}	13.4672	ar	5407.	3580.56	{3067., 4083.}	33.7792
33	R0	{1.55891, 8.28978, 1.}	{1.27748, 3.82593, 0.24}	{{1.22544, 3.07157, 0.}, {1.3618, 4.98002, 1.}}	{18.0528, 53.8477, 76.}	inf	446.	206.173	{127.427, 286.166}	53.773	0.354757	newly	409.	401.54	{291., 507.}	16.6161	0.0755674	Reff	1.30107	1.09661	{1.05281, 1.15973}	15.7149	ar	6239.	3892.49	{3223., 4781.}	37.6104
34	R0	{1.60764, 10.4729, 0.1}	{1.38789, 6.69996, 0.01}	{{1.38815, 6.71734, 0.}, {1.38815, 6.71734, 0.}}	{13.6691, 36.0257, 108.}	inf	658.	405.95	{352.782, 457.571}	38.3054	0.211782	newly	455.	437.05	{379., 491.}	7.93626	0.0360527	Reff	1.23097	1.09769	{1.09064, 1.10482}	10.8274	ar	6495.	5014.32	{4744., 5239.}	22.7972
35	R0	{1.48527, 5.74485, 1.}	{2.60679, 17.0995, 2.21}	{{2.46976, 17.202, 2.}, {2.86656, 18.9813, 3.}}	{75.9468, 200.036, 129.}	inf	223.	747.243	{665.328, 844.761}	236.944	0.521097	newly	365.	391.7	{337., 441.}	11.2438	0.0465263	Reff	1.37566	2.27402	{2.16255, 2.48145}	65.7019	ar	5811.	8938.72	{8825., 9316.}	54.7899
36	R0	{1.61457, 9.93364, 1.}	{1.25602, 3.4694, 0.01}	{{1.22544, 3.07157, 0.}, {1.28596, 3.98513, 0.}}	{22.2071, 65.0743, 99.}	inf	466.	166.977	{93.1079, 225.094}	64.168	0.464243	newly	363.	373.22	{223., 510.}	23.5647	0.105237	Reff	1.39273	1.12097	{1.09097, 1.14841}	19.5125	ar	6338.	3704.3	{2990., 4169.}	41.5541
37	R0	{1.47831, 7.08139, 0.1}	{1.70111, 7.60526, 0.88}	{{1.24751, 2.5364, 0.}, {2.40085, 15.146, 2.}}	{33.2425, 80.0108, 896.}	inf	307.	434.478	{61.085, 903.032}	107.156	0.447246	newly	351.	446.36	{206., 585.}	45.7721	0.197713	Reff	1.31939	1.47728	{1.12381, 2.00332}	28.2328	ar	5530.	5726.05	{3250., 8780.}	43.8696
38	R0	{1.73445, 9.8858, 0.1}	{1.31637, 4.35839, 0.27}	{{1.22544, 3.07157, 0.}, {1.28596, 3.98513, 0.}}	{27.5999, 65.3683, 354.}	inf	459.	181.333	{84.7662, 228.035}	68.7602	0.521165	newly	402.	329.47	{180., 456.}	24.6144	0.140596	Reff	1.53534	1.19824	{1.12149, 1.18554}	24.8974	ar	6842.	4051.06	{3049., 4480.}	43.7662
39	R0	{1.66621, 11.4824, 0.1}	{1.37988, 6.50522, 0.07}	{{1.3618, 6.25107, 0.}, {1.38815, 6.71734, 0.}}	{17.1848, 43.3462, 160.}	inf	585.	338.407	{279.43, 396.859}	42.1526	0.244652	newly	373.	393.2	{337., 448.}	11.7426	0.0493745	Reff	1.36629	1.16542	{1.15604, 1.17771}	14.7021	ar	6473.	4941.95	{4561., 5273.}	23.6529
40	R0	{1.60694, 9.95741, 1.}	{1.24613, 3.31491, 0.05}	{{1.22544, 3.07157, 0.}, {1.28596, 3.98513, 0.}}	{22.4533, 66.7091, 97.}	inf	536.	169.221	{103.166, 230.874}	68.4288	0.520319	newly	419.	386.97	{238., 512.}	22.2363	0.109789	Reff	1.35481	1.09627	{1.07225, 1.12829}	19.0832	ar	6625.	3609.61	{3075., 4170.}	45.5153
41	R0	{1.55128, 4.71911, 0.1}	{1.85043, 6.63796, 0.58}	{{1.47488, 4.00571, 0.}, {2.59133, 11.8519, 3.}}	{22.3352, 51.7423, 628.}	inf	143.	214.168	{98.9312, 391.2}	65.5883	0.211178	newly	305.	336.77	{227., 458.}	22.2131	0.0908611	Reff	1.48892	1.75868	{1.41627, 2.4311}	21.0183	ar	6141.	7096.01	{5674., 9060.}	20.6675
42	R0	{1.60063, 6.81118, 0.1}	{1.23166, 2.50497, 0.12}	{{1.19264, 2.19254, 0.}, {1.24751, 2.5364, 0.}}	{23.0512, 63.2227, 210.}	inf	336.	107.253	{32.3739, 186.775}	68.0795	0.57976	newly	454.	358.45	{105., 625.}	38.1388	0.246054	Reff	1.44265	1.1383	{1.09926, 1.16187}	21.0966	ar	6528.	3436.78	{2812., 3977.}	47.3532
43	R0	{1.56986, 5.44652, 0.1}	{1.64104, 5.47967, 0.39}	{{1.24751, 2.5364, 0.}, {2.95376, 15.278, 2.}}	{29.372, 68.5162, 450.}	inf	263.	262.113	{54.5674, 781.376}	78.4224	0.374453	newly	451.	410.51	{166., 573.}	27.51	0.153366	Reff	1.45887	1.50846	{1.17221, 2.59856}	26.3962	ar	6312.	5329.89	{3262., 9375.}	34.7574
44	R0	{1.47523, 8.07785, 0.1}	{1.41284, 5.61054, 0.26}	{{1.24751, 3.07157, 0.}, {1.44485, 6.00596, 1.}}	{6.39223, 33.8801, 324.}	inf	404.	285.766	{159.628, 366.909}	33.4014	0.192622	newly	403.	402.8	{310., 475.}	13.33	0.059328	Reff	1.27976	1.24129	{1.10144, 1.27523}	4.93495	ar	5692.	5039.11	{3569., 5654.}	14.3846
45	R0	{1.6417, 7.69429, 0.1}	{2.21483, 12.203, 1.05}	{{1.44762, 4.00571, 0.}, {2.60082, 15.3681, 2.}}	{39.363, 71.5708, 978.}	inf	406.	787.839	{311.322, 1053.02}	103.067	0.303247	newly	444.	569.06	{485., 638.}	28.7568	0.107068	Reff	1.44486	1.85358	{1.28776, 2.13476}	32.4244	ar	6735.	8058.28	{5422., 9084.}	27.8295
46	R0	{1.38865, 5.83473, 0.1}	{1.24668, 3.24605, 0.02}	{{1.22544, 2.5364, 0.}, {1.28596, 3.98513, 0.}}	{10.2238, 44.3667, 116.}	inf	250.	147.181	{78.9638, 206.032}	41.7502	0.275133	newly	337.	348.78	{197., 485.}	27.0742	0.132625	Reff	1.23465	1.12119	{1.09904, 1.15015}	9.18966	ar	5233.	3541.98	{2897., 4125.}	32.3145
47	R0	{1.45289, 6.87903, 0.1}	{1.25376, 3.42112, 0.02}	{{1.22544, 3.07157, 0.}, {1.28596, 3.98513, 0.}}	{13.7058, 50.2674, 116.}	inf	272.	171.112	{117.835, 238.368}	37.4379	0.221265	newly	303.	391.77	{256., 512.}	36.1683	0.13108	Reff	1.28537	1.12182	{1.09403, 1.15105}	12.7241	ar	5379.	3750.78	{3204., 4226.}	30.2699
48	R0	{1.69416, 9.4831, 0.1}	{1.93883, 11.4252, 0.03}	{{1.25474, 3.26445, 0.}, {2.59755, 18.2464, 0.}}	{30.4133, 60.8817, 124.}	inf	453.	495.575	{134.992, 788.269}	53.4726	0.266369	newly	442.	368.37	{284., 436.}	17.405	0.0886977	Reff	1.51407	1.71197	{1.15635, 2.20957}	27.3653	ar	6925.	7024.05	{3657., 9003.}	28.7788
49	R0	{1.47843, 7.16286, 0.1}	{1.39311, 4.73636, 0.67}	{{1.24751, 2.5364, 0.}, {2.03251, 13.7179, 3.}}	{19.4034, 68.4502, 728.}	inf	357.	254.35	{68.1899, 689.463}	66.9461	0.4127	newly	403.	439.57	{201., 668.}	30.1563	0.134588	Reff	1.2988	1.23148	{1.11216, 1.70808}	16.7162	ar	5449.	4484.28	{3146., 8005.}	35.7071
50	R0	{1.68843, 5.72427, 0.1}	{1.45937, 3.80182, 0.}	{{1.24751, 2.5364, 0.}, {1.53501, 4.06661, 0.}}	{13.9374, 33.6225, 100.}	inf	329.	243.859	{128.287, 352.346}	29.556	0.175868	newly	521.	589.99	{370., 753.}	28.4395	0.117987	Reff	1.53732	1.34051	{1.15971, 1.4074}	13.0961	ar	6800.	5449.47	{3741., 6143.}	20.3134
